# *Rhantus fengi* sp. n. from Xizang, China, and notes on *Laccoporus nigritulus* (Gschwendtner) (Coleoptera, Dytiscidae)

**DOI:** 10.3897/zookeys.94.1161

**Published:** 2011-05-03

**Authors:** Shuang Zhao, Fenglong Jia, Michael Balke

**Affiliations:** 1 Institute of Entomology, Life Science School, Sun Yat-sen University, Guangdong, China; 2 Zoologische Staatssammlung München, Münchhausenstr. 21, D-81247 München, Germany and GeoBio-Center, Ludwig-Maximilians-University, Munich, Germany

**Keywords:** China, Tibet, high altitude, *Rhantus*, *Laccoporus*, Dytiscidae, new species, new synonymy

## Abstract

*Rhantus fengi* **sp. n.** from Mount Sejila, Xizang, China is described and illustrated. *Laccoporus nigritulus* (Gschwendtner, 1936) is redescribed and illustrated; *Laccoporus viator* Balfour-Browne, 1939, **syn. n.** is established as its junior subjective synonym.

## Introduction

Xizang Autonomous Region is one of the largest provinces of China. The environment varies considerably with different elevation, but rich aquatic resources such as natural lakes and wetlands are abundant up to 5500 meters altitude. Yet, the diving beetle fauna of Xizang remains comparably little known due to the inaccessibility of many areas. Nilsson (1995) reported only eighteen species of diving beetle from “Tibet”, or the Tibetan Plateau, an area that includes parts of Sichuan and Xizang as well as Qinghai, Gansu and Yunnan. Subsequently, another six species were reported from “Tibet”, mainly from Qinghai and Sichuan (Toledo and Mazzoldi 1996; Toledo 1998; Fery 2003; Brancucci and Hendrich 2006, 2008).

In 2009, the first two authors and Shuai Jiang collected a series of specimens of *Rhantus* Dejean, 1833 in Xizang, close to Mt. Nanjiabawa, which could not be assigned to any known species of the genus. This species is here described as new to science. In addition, the very rarely collected *Laccoporus nigritulus* (Gschwendtner, 1936) is redescribed based on specimens in the insect collection of Sun Yat-sen University (Guangzhou, Guangdong, China).

## Material and methods

Morphological terminology largely follows Nilsson and Holmen (1995), Balke (1993) and Miller and Nilsson (2003). Photographs were taken using a Zeiss Axioskop 40 compound microscope and an Olympus SZX7 stereomicroscope combined with AutoMontage software; with a Leica M205C equipped with Zeiss Progres Camera (detail of tarsus and detail of surface sculpture of *Rhantus fengi* presented in the supporting online resources) and with a Leica Photar 25 mm on a bellows attached to Nikon D700 and using Helicon Focus stacking software (dorsal habitus of *Rhantus fengi* and *Rhantus gogonensis*).

**Codens**

BMNH	Natural History Museum, London, UK

CGC	Collection of G. Challet, California, USA

CHF	Collection H. Fery, Berlin, Germany; property of the Natural History Museum Vienna, Austria

MTD	Museum für Tierkunde, Dresden, Germany

NMPC	Národní Museum, Prague, Czech Republic

SYSU	Sun Yat-sen University, Guangzhou, China

ZSM	Zoological State Collection Munich, Germany

ZSIC	Zoological Survey of India in Calcutta, India

### Online Resources

We have created species pages for the species treated herein on Species ID (http://species-id.net/wiki/Main_Page) where we provide additional illustrations as well as high-resolution versions of the images used here.

## Systematics

### Rhantus fengi sp. n.

urn:lsid:zoobank.org:act:F0EC468E-CA7D-4A0C-B722-65A0616873FA

http://www.species-id.net/wiki/Rhantus_fengi

Figs 1–8 

#### Type locality:

China: Xizang, Mount Sejila, 29°37'N, 94°37'E.

#### Type material.

**Holotype** ♂ CHINA: Xizang, Mount Sejila, altitude 4200 m, 16.viii.2009, leg. Fenglong Jia [translation; labeled in Chinese] (SYSU); Paratypes (47 exs): 12 exs, same data as holotype (SYSU; 3 exs in CGC; 2 exs in ZSM); 4 exs same data as holotype but 12.viii.2009 (SYSU); 7 exs same data as holotype but 15.viii.2009 (SYSU); 4 exs same data as holotype but 17.viii.2009 (SYSU); 4 exs same data as holotype but 18.viii.2009 (SYSU); 3 exs same data as holotype but 13.–15.viii.2009 (SYSU); 2 exs Xizang, Bayi Town, Biological Study Base of SYSU, altitude 4200 m, vii.–ix.2009, leg. Shuai Jiang (SYSU); 4 exs Xizang, Mount Sejila, altitude 4100 m, vi.2009, leg. Shuang Zhao (SYSU).

#### Diagnosis.

General appearance of beetle rather dark, epipleuron black.

#### Description.

**Figures 1–8.**
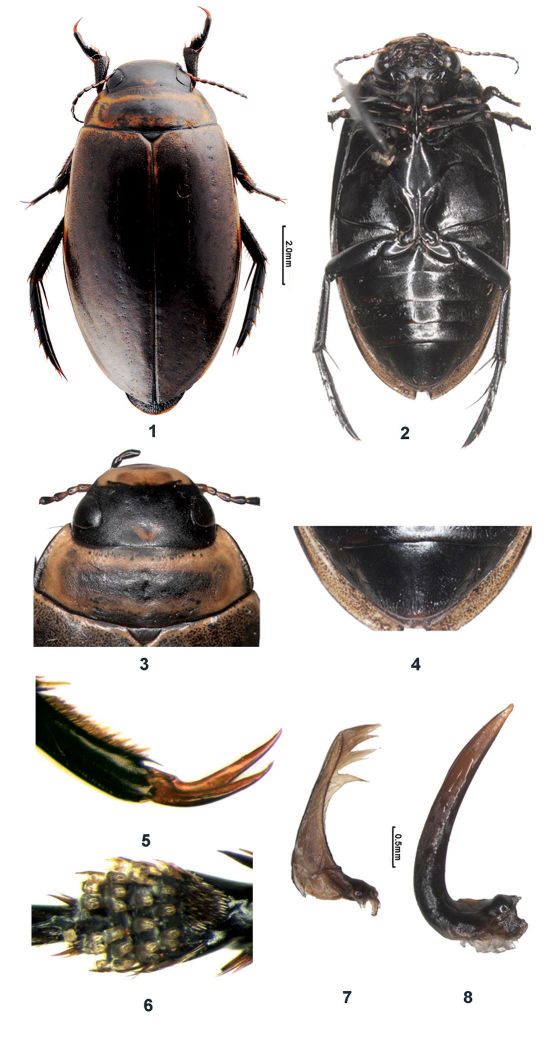
*Rhantus fengi* sp. n. **1** habitus, dorsal view **2** habitus, ventral view **3** head and pronotum, dorsal view **4** ventral view of 6th ventrite **5** male protarsal claws **6** ventral view of male protarsomeres 1–4 **7** right paramere, inner view **8** median lobe of aedeagus, lateral view.

Body elongate-oval (Fig. 1).

#### Measurements.

Body length 13.2–14.1 mm, width 6.2–6.5 mm.

#### Colour.

Head black, with orange triangular mark on frons and contrasting yellow clypeus (Fig. 3). Scape and pedicel yellow, antennomeres 2–5 piceous with yellow base, antennomeres 6–11 piceous. Maxillary and labial palps piceous. Pronotum yellow to yellowish brown, with a wide transverse black medial maculation that not reaching anterior and posterior margin; anterior and posterior margins with black bands rather broad medially that become lighter laterally, sometimes becoming thin laterally, lateral margin black except anterior angle, near anterior and posterior margins usually with somewhat regular black spots. Elytra yellow to yellowish brown, disc with very dense black speckles giving elytra dark appearance; lateral margin, base of elytra and near suture with yellow band, suture black; epipleura black. Ventral surface and legs black (Fig. 2), trochanters with yellowish base, pro- and mesotarsal claws somewhat dark brown.

#### Surface sculpture.

Head with irregular polygonal meshes and fine punctation; meshes rather elongated and punctures coarser behind posterior margins of eyes; along inner face of eye with series of coarse setiferous punctures. Pronotum with irregular polygonal meshes and fine punctuation; along anterior and posterior margins with rows of coarse punctures, interrupted posteromedially; lateral portion distinctly depressed, without microreticulation, with broad rim (or border) reaching anterior angle; posterior angle broadly rounded. Elytra with irregular polygonal meshes and fine microreticulation best visible laterally and posteriorly (at 50×); each elytron with five rows of coarse setiferous punctures being somewhat irregular except in row 1 (sutural row) and very regular row immediately along lateral margin; elytral disc with similar meshes and fine punctures as on pronotum, mircroreticulation faint, hardly visible. Metaventrite (“metasternal wings”) broad laterally. Metafemur without group of setae on posteroexternal angle. Pro- and mesotarsomeres with claws gently curved; outer protarsal claw slightly shorter than inner one (Fig. 5) and inner mesotarsal claw distinctly shorter than outer one; inner hindclaw twice as long as outer one. Abdominal ventrites 4 and 5 with a transverse rows of coarse setiferous punctures; ventrite 6 rugose with stronger punctures and setae laterally, more or less depressed and smooth posteromedially.

#### Male.

Abdominal ventrite 6 posteriorly emarginate, with fine wrinkles laterally. Pro- and mesotarsomeres 1–3 clearly expanded laterally, with four rows of stalked suction discs ventrally (Fig. 6). Number of discs per transverse row: 5 discs (on tarsomere 1) - 5 discs (second row on tarsomere 1) – 5 discs (on tarsomere 2) - 5 discs (on tarsomere 3). Parameres with dense and long setation, some setae distally trumpet-shaped (Fig. 7). Median lobe of aedeagus as in Fig. 8.

#### Female.

Abdominal ventrite 6 simply rounded posteriorly (Fig. 4), pro- and mesotarsomeres 1–3 not expanded laterally, without stalked suction discs.

#### Remarks.

**Figure 9.**
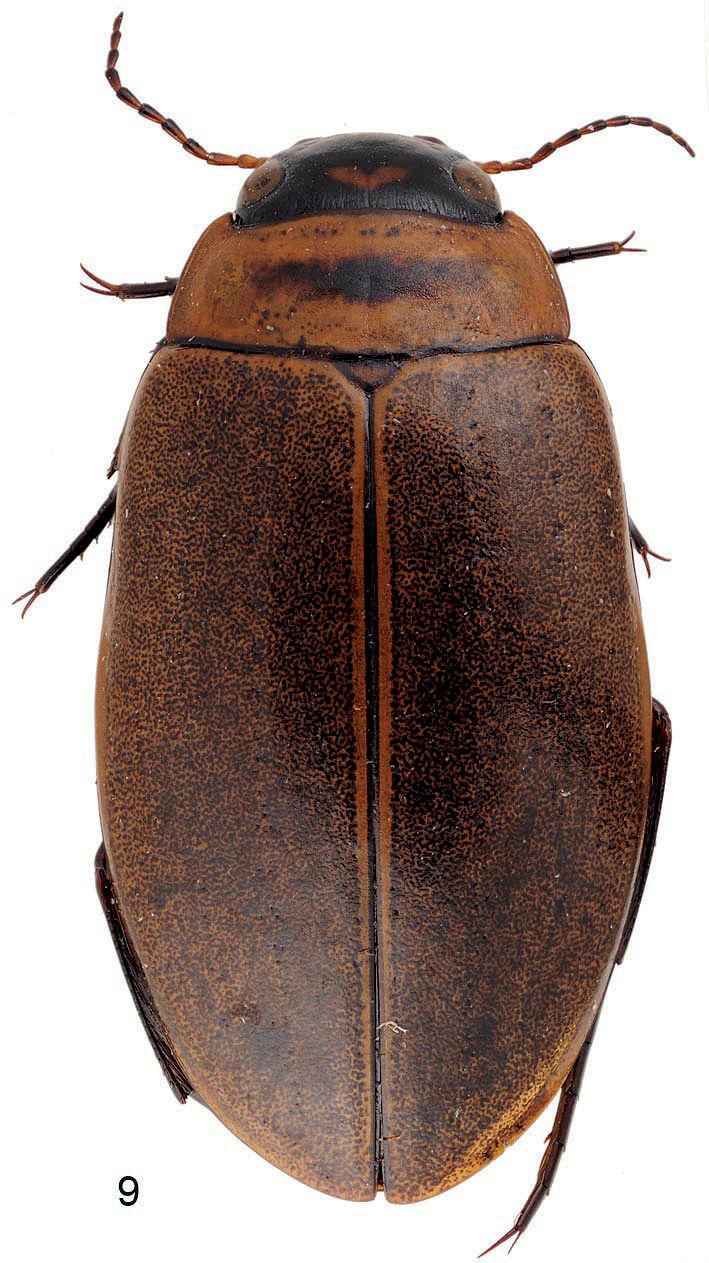
*Rhantus gogonensis* habitus.

The elytral sculpture of polygonal meshes and microreticulation as well as the parameres with some trumpet shaped setae, place this species in the *Rhantus suturalis* group (Balke 1993). The species is well characterized by its black epipleura; this is shared only with the Bhutanese species *Rhantus gogonensis* Wewalka, 1975 (Fig. 9) which is only known from the female holotype (Bhutan: Sha Gogona; http://www.species-id.net/wiki/Rhantus_gogonensis). *Rhantus fengi* differs from the latter by the darker body, pronotum not narrower than the base of elytra; pronotum distinctly impressed laterally; head, pronotum and elytra with very dense fine punctures, and black tarsi and antenna.

#### Etymology.

The species is named in honour of Hsiao-Tang Feng, a pioneer in the Chinese Dytiscidae studies.

#### Habitat.

Collected from a branch of a small stream with fine sand on bottom. The water of the stream was about 10–15 cm deep and slowly flowing.

### Genus Laccoporus

J. Balfour-Brown, 1939

http://www.species-id.net/wiki/Laccoporus

#### Notes.

This genus is very similar to *Laccophilus* Leach, 1815, with the major difference being presence of apically acute metatibial spurs in *Laccoporus* (apically bifid in *Laccophilus*). However, this character has been shown to be reversible in *Laccophilus*, even though rarely so (Balke et al. 1997). We suspect that a study of DNA sequence data will reveal *Laccoporus* in a position within *Laccophilus*.

#### Type species:

*Laccoporus viator* Balfour-Browne, 1939: 104, by original designation.

### Laccoporus nigritulus

(Gschwendtner, 1936)

http://www.species-id.net/wiki/Laccoporus_nigritulus

Figs 10–15

Laccophilus apicicornis  var. *nigritulus*Gschwendtner 1936: 367.
Laccoporus nigritulus  (Gschwendtner): Vazirani 1970: 563–564 (new combination, new status); Nilsson 2001: 253.
Laccoporus viator Balfour-Browne 1939: 104; Nilsson 2001: 253; new synonymy.
Laccophilus  sp.: Nilsson 1995: 71.

#### Type localities:

*Laccophilus apicicornis* var. *nigritulus*: China: Tibet, Te-ring Gompa; *Laccoporus viator*: China: Tibet, Gyangtse.

#### Type material:

*Laccophilus apicicornis* var. *nigritulus*: 10 syntypes, Te-ring Gompa. 14,000 ft. Tibet (F.H. Stewart) in ZSIC [not studied]. Nilsson (2001) mentioned a lectotype designation by Vazirani (1970), but Vazirani mentioned that he studied the holotype and one paratype, the type status is therefore rather unclear and we could not access types from ZSIC to clarify the situation.

*Laccoporus viator*: Holotype, „allotype” and 10 other paratypes in BMNH. We studied 2 *♂♂* paratypes labelled: „Co-type”, „Gyangtse. / 13,000 ft. / June 1904. / Tibet Expedit. / H.J. Walton. / 1903–172.”, „Laccoporus / viator. B-B. / Co-type.”.

#### Other material examined:

20 exs (SYSU): 2 *♂♂* **CHINA:** Xizang, Dingri (Tering), 4300 m, 2.vi.1974, leg. Xuezhong Zhang, Academia Sinica; 1 ♀ the same data as male; 1 ♀ Xizang, suburb of Rikaze, 3826 m, 20–23.vii.1986, leg. Geqiu Liang; 5 *♂♂*, 3 ♀♀ Xizang, Rikaze, 3862 m, 4.viii.1986, leg. Geqiu Liang; 1 *♂* the same data as the former, with a label „Laccophilus indicus ?”; 4 *♂♂*, 3 ♀♀ Xizang, Mozhugongka County, 7.viii.1986, leg. Geqiu Liang; 8 exs (MTD, NMPC, CHF) „Tibet, Yamtso-ufer bei / Nagartze, N28°58'31,9 / E90°24'6,0; 4450mNN; / 29.VII. 1998; leg. O.Jäger”, „Laccoporus nigritulus / (Gschwendtner) / Fery det. 1999"; 11 exs (MTD, CHF) „Tibet, Tingriebene /  N28°34'39,7/E86°36'52,7; 4400 m / 3.-5.VIII. 1998, lg. Jäger“, „Laccoporus nigritulus / (Gschwendtner) / Fery det. 1999”.

#### Redescription.

Measurements. Length 4.5–5.0 mm, width 2.5–2.6 mm (paratypes of *Laccoporus viator*: length 4.5–4.8 mm, width 2.3–2.6 mm).

#### Colouration

**Figures 10–15.**
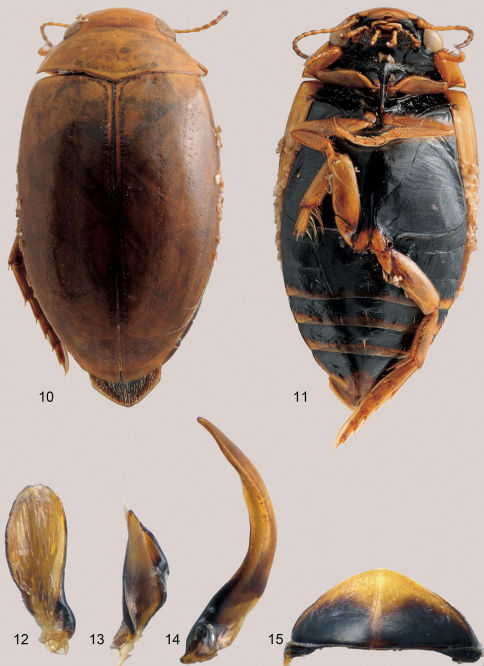
*Laccoporus nigritulus* male **10** habitus, dorsal view **11** habitus, ventral view **12** left paramere **13** right paramere **14** median lobe of aedeagus, lateral view **15** ventral view of 6th ventrite.

**(Figs 10, 11, 15).** Dorsum uniformly yellow brown. Head yellowish brown, with one short dark brown line near antennae. Antenna with antennomeres 1–4 and base of antennomeres 5–6 yellow brown, antennomeres 7–11 andapical parts of antennomeres 5–6 dark brown. Maxillary palpomeres yellow brown, apical palpomere apically dark. Labial palpomeres yellow brown, apical palpomere dark. Pronotum sometimes paler with darker median, anterior and posterior transverse bands. Elytra clearly darker than pronotum, but with the same color at base as pronotum. Ventral surface of head and thorax black. Abdomen black, ventrites 3–6 yellow brown along posterior margins (e.g. Fig. 15). Legs yellow brown with coxae black, posterior half of metacoxal process yellowstructures(see http://www.species-id.net/wiki/Laccoporus_nigritulus for high resolution images of surfaces). Head with surface sculpture consisting of small irregular polygonal meshes, with some sparse coarse and shallow punctures in posterior half. Meshes somewhat stronger on posterior portion. Clypeus with transverse series of large punctures present anteriorly. Labrum with very deep excavation, surface with short transverse meshes, anterior notch with dense and short white setae. Antenna filiform, not dilated. Pronotum more or less depressed laterally, without lateral bead, with small irregular polygonal meshes, sometimes very tiny punctures detectable. Posterior corner almost rectangular. Elytra with base as wide as posterior portion of pronotum, surface with irregular polygonal meshes in size and shape, sculpture more or less stronger than that on pronotum, tiny punctures very sparse and clearly detectable. Prosternal process thin and long,bisinuate in lateral view, almost reaching posterior margin of mesocoxae. Metaventrite medially with some coarse and strong punctures behind mesocoxae. Metacoxae and metasternum strongly and irregularly scratched, with dense longitudinal sculptures and sparse tiny punctures. Pro- and mesofemora with tuft of long setae at basal portion posteriorly. Metafemora with fine and dense sculpture and tiny punctures. Metatibia with inner spur longer than outer one. Pro- and mesotarsi smooth. Metatarsi smooth, with fine sculpture dorsally. First tarsomere of metatarsi almost as long as following two tarsomeres combined. Claws normally curved, protarsal claws longer than metatarsal claws. Metatarsomere 1 almost as long as metatarsomeres 2–3 combined. Claws regularly curved, proclaws longer than metaclaws.

#### Male.

Protarsus not dilated. Median lobe and parameres as in Figs 12–14.

#### Distribution.

China: Xizang.

#### Remarks.

Genus *Laccoporus* was erected by Balfour-Browne (1939) with *Laccoporus viator* Balfour-Browne, 1939 as the type species. This author had no knowledge about *Laccophilus apicornis* var. *nigritulus* Gschwendtner, 1936. The latter taxon was transferred to *Laccoporus* and raised to species status by Vazirani (1970) who studied two syntypes housed in ZSIC. Vazirani (1970) had doubts that *Laccoporus viator* and *Laccoporus nigritulus* were different species, but he could not study Balfour-Browne’s types. We have no access to specimens in ZSIC, but Vazirani (1970) provided an illustration of the ventral side of the median lobe of *Laccoporus nigritulus*, which agrees with our specimens.

**Figure 16.**
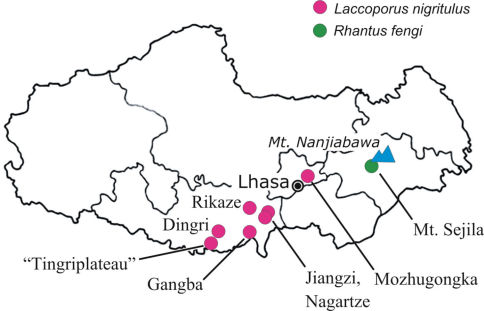
Xizang Autonomous Region, collecting localities of *Rhantus fengi* and *Laccoporus nigritulus* and notable geographic localities for orientation. Dingri = Te-ring (type locality of *Laccoporus nigritulus*); Gangba = Gompa; Jiangzi = Gyangtse (type locality of *Laccoporus viator*).

The types of *Laccoporus viator* are from Gyangtse (Jiangzi). The types of *Laccoporus nigritulus* were collected from “Te-ring” (Dingri) and “Gompa” (Gangba). The distance between these sites is only about 100–120 km (Fig. 16). The two latter sites are not far (about 90–120 km respectively) from Rikaze where some of our newly studied specimens originate from. The altiude of these three sites is 3900–4300 m.

Zeng (1989) described “*Laccophilus thibetanus* sp. n.” from Rikaze, Xizang in her PhD thesis (unpublished, in Chinese) (in the corrected thesis version, the name was changed to *Laccoporus xizangensis* sp. n. by herself, possibly acknowledging that Xizang is a more precise reference to the type locality than “Tibet”). Nilsson (1995: 71) recorded a “*Laccophilus* sp.” and stated that it is based on “*Laccophilus tibetanus* Zeng, 1989: 4 (nom. nud., Tibet)”. We found the specimens that have the same locality data as recorded by Zeng (1989) in SYSU. Although there is no specific name and no type designation attached to these specimens, we are confident that Zeng (1989) based her unpublished *Laccophilus xizangensis* on these specimens. All the specimens are conspecific with *Laccoporus nigritulus*.

## Supplementary Material

XML Treatment for Rhantus fengi

XML Treatment for Laccoporus

XML Treatment for Laccoporus nigritulus

## References

[B1] Balfour-Browne J (1939) A contribution to the study of the Dytiscidae.–I. (Coleoptera, Adephaga). The Annals and Magazine of Natural History (11) 3:97-114

[B2] Balke M (1993) Taxonomische Revision der pazifischen, australischen und indonesischen Arten der Gattung *Rhantus* Dejean, 1833 (Coleoptera: Dytiscidae). Koleopterologische Rundschau 63:39-84

[B3] Balke M Larson DJ Hendrich L (1997) A review of the New Guinea species of Laccophilus with notes on regional melanism (Coleoptera: Dytiscidae). Tropical Zoology 10 (2):295-320

[B4] Brancucci M (1983) A new genus of the subfamily Laccophilinae (Coleoptera, Dytiscidae). Aquatic Insects 5: 251–254 doi:10.1080/01650428309361151

[B5] Brancucci M Hendrich L (2006) A new high-altitude *Ilybiosoma* Crotch, 1873 from Tibet, as an example of a Palaearctic–Afrotropical disjunction (Coleoptera, Dytiscidae). Aquatic Insects 28(2): 131–138 doi:10.1080/01650420600684634

[B6] Brancucci M Hendrich L (2008) 5100 m above sea level: *Agabus joachimschmidti* sp.n. and notes on other high altitude diving beetles from Tibet and Bhutan (Coleoptera, Dytiscidae). Zootaxa 1825:51-58

[B7] Fery H (2003) Taxonomic and distributional notes on *Hygrotus* Stephens, with emphasis on the Chinese fauna and a key to the Palearctic species. In: Jäch MA Ji L (Eds) Water beetles of China, Vol. 3. Zoologisch-Botanische Gesellschaft in Österreich and Wiener Coleopterologenverein, Wien, 133–193

[B8] Gschwendtner L (1936) Interessante und neue Schwimmkäfer des Indischen Museums in Calcutta. Records of the Indian Museum 37 (1935) (3): 365–374

[B9] Miller KB Nilsson AN (2003) Homology and terminology: Communicating information about rotated structures in water beetles. Latissimus 17:1-4

[B10] Nilsson AN (1995) Noteridae and Dytiscidae: Annotated checklist of the Noteridae and Dytiscidae of China (Coleoptera). In: Jäch MA Ji L (Eds) Water beetles of China, Vol. 1. Zoologisch-Botanische Gesellschaft in Österreich and Wiener Coleopterologenverein, Wien, 35–96

[B11] Nilsson AN (2001) Dytiscidae. – World Catalogue of Insects. Vol. 3. Apollo Books, Stenstrup, 395 pp.

[B12] Toledo M Mazzoldi P (1996) Two new Dytiscidae from south-western China. Natura Bresciana, Annuario del Museo Civico di Storia Naturale di Brescia 30 (1994):237-245

[B13] Toledo M (1998) Dytiscidae: II. The genus *Nebrioporus* Régimbart, 1906 in China (Coleoptera). In: Jäch MA Ji L (Eds) Water beetles of China, Vol. 2. Zoologisch-Botanische Gesellschaft in Österreich and Wiener Coleopterologenverein, Wien, 69–91

[B14] Vazirani TG (1970) On *Laccoporus nigritulus* (Gschwendtner) comb. n. (Coleoptera: Dytiscidae). Current Science 39 (24):563-564

[B15] Wewalka G (1975) Ergebnisse der Bhutan-Expedition 1972 des Naturhistorischen Museums in Basel. Coleoptera: Fam. Dytiscidae, unter Berücksichtigung einiger Arten aus benachbarten Gebieten. Entomologica Basiliensia 1:151-165

[B16] Zeng H (1989) Taxonomy of Chinese Dytiscidae in the museums of China. PhD thesis, Department of Biology, Sun Yat-sen University, Guangzhou

